# The Protective Effects of Beef and Yeast Extracts and Copper Acetate in the Diet Against Rat Liver Carcinogenesis by 4-Dimethylaminoazobenzene

**DOI:** 10.1038/bjc.1964.91

**Published:** 1964-12

**Authors:** G. Fare


### THE PROTECTIVE EFFECTS OF BEEF AND YEAST EXTRACTS AND COPPER ACETATE IN THE DIET AGAINST RAT LIVER CARCINOGENESIS BY 4-DIMETHYLAMINOAZOBENZENE

G. FARE

*From the Cancer Research Laboratories, Department of Pathology, Medical School, Birmingham, 15*

Received for publication July 3, 1964

IT has long been known that the composition of the diet influences azo dye carcinogenesis in the liver of the rat and that the tumour induction time may be lengthened or shortened for any particular dye concentration by such factors as the content of protein, fat and vitamins in the diet. For example, riboflavin is a good protective agent (Kensler *et al.*, 1941) which probably acts by accelerating the rate of destruction of the carcinogen by the azo dye reductase system, of which flavin-adenine dinucleotide is the coenzyme (Miller and Miller, 1953).

Following earlier reports that the addition of copper salts to the diet afforded some protection, King, Spain and Clayton (1957) reported that a diet high in copper diminished the carcinogenicity of 3′-methyl-4-dimethylaminoazobenzene, one of the most potent of the azo dye carcinogens. They ascribed an appreciable part of this protection to *in vitro* destruction of the dye in the diet accelerated by the copper ; thus their results were due to feeding a lower amount of carcinogen.

In these laboratories, using the weaker carcinogen 4-dimethylaminoazobenzene (DMAB) at a concentration of 0·09 per cent in a maize diet, the addition of 0·5 per cent of cupric oxyacetate has been observed to give consistently a good degree of protection, and it has been shown that there is no detectable destruction of the dye in our maize + dye + copper acetate diet even after several months storage (Howell, 1958). It has been suggested (Fare, 1964) that protection is associated with the ability of copper to bind with liver protein thereby competing with the carcinogen for the available binding sites.

Although it is rare for a rat to develop liver cancer when the copper salt is given together with DMAB, complete protection is not achieved and this paper describes experiments carried out in an attempt to obtain this by supplementing the copper salt with riboflavin. Experiments are also included which were designed to assess the relative efficacies of these two chemicals as protective agents. Riboflavin was used in the form of two extracts of biological material, a yeast autolysate and a mixture of three extracts of bovine tissues. These materials contained other substances of possible prophylactic value as well as a suitable content of the vitamin.

### MATERIALS AND METHODS

*Dietary ingredients.*—All the experimental diets were based on maize meal. This is of poor nutritive value for the rat because of its deficiency in lysine, tryptophan, calcium, phosphorus and several vitamins ; it has a high content of fat and starch. In our experience, rats do not thrive on a maize diet and consequently for two consecutive days each week a proprietary rat cube (Thomson diet) is substituted to supply the factors missing in the maize. Under these conditions, our animals grow normally and it is very unusual for a rat to die from infection, malnutrition or other adventitious cause no matter what liver-damaging agent is included in the diet, and this dietary regime is therefore used routinely in these laboratories for experiments of this type.

DMAB was obtained from British Drug Houses Ltd., and cupric oxyacetate hexahydrate from Hopkin and Williams Ltd. Both chemicals were used without purification.

An unflavoured yeast extract manufactured by the autolysis of brewer's yeast with salt was given by Marmite Ltd., London, E.C.3.

The three animal extracts, given by Oxo Ltd., London, S.E.1. were normal commercial products. They were always fed to the animals in combination in the proportions 80 per cent of " beef stock " (a steam extract of meaty bones), 12 per cent of " beef extract " (fresh lean meat extracted with boiling water) and 8 per cent of " beef powder " (heat dried beef muscle).

*Preparation of diets.*—Nine diets, assigned the letters A–I for easy reference, were used.

The two control groups were :

A Maize only, to give the normal values, and

B Maize + 0·09 per cent DMAB to give the values obtained without a protective agent in the diet.

The remaining seven diets all contained 0·09 per cent of the dye with the respective protective agents :

C 0·5 per cent copper acetate (CuAc)

D Yeast extract

E Beef extracts

F 0·5 per cent CuAc + beef extracts

G 0·5 per cent CuAc + yeast extract

H 0·5 per cent CuAc + yeast + beef extracts

I Yeast and beef extracts

Twenty female outbred albino rats, aged 3–4 months, were used in group A, 27 in B, 28 in C and 6 in each of the remaining groups. Details of housing and of preparing the maize plus dye and copper acetate diets have been given previously (Howell, 1958). Aqueous " solutions " of the two extracts were prepared every 10 days and stored at 4° C. in stoppered brown bottles. The meat extract was always shaken before use as the " beef powder "component was insoluble. Each day, the relevant dry diet was placed in the trough for each cage and the appropriate aqueous solutions of the extracts were added. In every case, the volume added was such that each rat received an extra 40 $\mu$g. of riboflavin. In those diets where extract solutions were not added, tap water was used to moisten the food to prevent scattering in the bedding which would have made assessment of food consumption difficult. At the start of the experiment, the rats ate on average 10 g. of dry diet daily, but this rose to 25 g. per day later in the experiment. In every case, the amount of food offered was slightly in excess of the daily requirement, and in the groups given the copper salt each rat received from 17 to 40 mg. of extra cupric ion per day depending upon the amount of food consumed.

*Plan of experiment.*—Previous work using copper as a protective agent and DMAB as carcinogen has shown 5 simple criteria of the degree of cancer involvement in the liver (Fare and Woodhouse, 1963*a*, 1963*b*). There is the obvious one of visible liver damage, while the change in the ratio of body weight to liver weight affords a good idea of tumour progression. The decrease in nitrogen content and in succinoxidase activity and also the increase in copper storage in the liver may all be used as biochemical criteria of liver damage by DMAB. All five criteria were applied for evaluating and comparing the degree of protection in the experiments described here.

Single rats were killed from the various groups at approximately regular intervals during treatment for up to 400 days of diet feeding. The rats in any one cage were identifiable by their ear clips so that an animal could be selected at random by the blind selection of one numbered disc out of six before the rats themselves were inspected. In this way biased selection was eliminated, and the rat killed could be taken as typical of its cage mates at that time allowing, of course, for individual variation.

A rat to be killed was first starved for 16 hours to give a reasonably standard liver glycogen content, weighed, killed with ether and the liver immediately removed, washed with cold water, weighed, chopped up with scissors and minced in a chilled, hand-operated mincer fitted with a 1 mm. stainless steel mesh to retain connective and vascular tissue. An accurately weighed sample (about 100 mg.) of the " parenchymatous " pulp was homogenised in physiological saline (10 ml.) and stored at 4° C.

Aliquots were assayed for nitrogen, copper and succinoxidase activity by methods described previously (Fare and Woodhouse, 1963*a*). The parameters were all expressed in terms of weight of parenchymatous liver pulp.

### RESULTS

All the rats grew satisfactorily and maintained good health on the diets and there were no deaths from adventitious causes. In no case did a rat have to be killed for reasons of ill-health, and consequently all killings were prompted solely by experimental convenience. Groups which developed tumours were killed earlier than the better protected groups for obvious reasons although one rat from each group was killed after 350 days so that direct comparisons could be made at this arbitrary time. The times between which the rats were killed in each group may be found in Table I.

The amounts of food consumed in the various groups did not differ, but the rats given one or both of the extracts usually consumed their food at an earlier time each day than did the animals not given the extracts.

Table I shows the times at which post-mortem examination first revealed liver damage and liver tumours visible with the naked eye. Since animals were selected for killing by a random process, it sometimes happened that a later killing from the same cage disclosed a normal liver. The times at which the last normal liver was seen in each group are therefore also included in the table.

The early liver damage produced by a diet containing DMAB is usually seen as a darkening and roughening of the surface, and these were the only changes found in the group H animals during 400 days treatment. Later changes in the liver are an increase in size and the development of small black nodules, still

TABLE I.—*Times at which Liver Damage and Tumours were First Discovered*

| Group | Time on diet (days) | Liver damage First seen (days) | Liver damage Last Normal liver seen (days) | Liver tumours First seen (days) | Liver tumours Last liver seen free from tumour (days) |
|---|---|---|---|---|---|
| B | 50–350 | 95 | 130 | 185 | 205 |
| C | 80–390 | 230 | 285 | | 390 |
| D | 40–385 | 115 | 90 | 285 | 180 |
| E | 40–350 | 135 | 40 | 295 | 205 |
| F | 145–385 | 240 | 190 | | 385 |
| G | 145–380 | 225 | 190 | | 380 |
| H | 145–405 | 285 | 315 | | 405 |
| I | 145–385 | 225 | 250 | 350 | 250 |

Rats were killed singly at various times between 40 and 405 days after treatment started. The times of the first and last killings for each group are given in column 2. A rat was selected at random for each killing, and the post-mortem findings were assumed to be typical of the dietary group. Individual variation gives rise to normal livers being found after longer periods of treatment than the first abnormality to come to autopsy, e.g. groups B, C, H and I.

without the appearance of tumours. These changes were found in the other three groups on diets containing copper (C, F and G).

All the other groups given DMAB produced tumours which were found after 6 months when the carcinogen was given alone, after 10 months when either of the extracts was given in addition and after 11 months when both extracts were given. Tumours produced arose either from the parenchyma (true liver cell tumours or hepatomas) or from bile duct tissue (cholangioma and cystadenoma) and all three types were found in rats from each group that produced tumours. Their relative abundance was unchanged when one or both extracts were given indicating that protection was being given equally against the induction of all three types of tumour.

In Fig. 1, the post-mortem ratio of body weight to liver weight is given for each group after an arbitrary time of 350 days. The maize controls had a mean value of 26·8, standard deviation 1·2, and it can be seen that the rat given the dye alone for this period had a ratio of only 4·9. The protective diets gave rise to an intermediate range of values.

The nitrogen values are given in Fig. 2. After only 60 days, the rat given DMAB alone had fallen below the normal range of liver nitrogen content, and after 350 days the value was 1·85 as opposed to the normal value of 3·15. When either extract was fed, the value remained in the normal range for about 110 days and then fell gradually as before. The combined extracts occasioned a longer delay but the value was sub-normal after about 210 days, and copper acetate with or without either extract delayed the fall by about 280 days.

Once the values had fallen below normal, the continued falls were parallel in every case. Protection was therefore concerned with the lengthening of the induction period ; the process once started was unaffected. Rats given diet H gave values in the normal range throughout.

The copper contents of the livers from animals on diets which were not supplemented with copper are given in Fig. 3. The values lie on a smooth curve corresponding to those obtained by administering the carcinogen alone and show an increase of 40 per cent above normal after 400 days. Increased copper storage in the liver would therefore seem to be a reaction simply to DMAB administration irrespective of the amount of liver damage produced where this is controlled by altering the riboflavin content of the diet.

In other words, the level of copper storage in the liver is not a criterion of the degree of tumour progression in that organ.

FIG. 1.—Ratio of body weight to liver weight, post mortem after treatment for 350 days.

All the copper-supplemented diets produced the same increase in liver copper content, between 35 and 45 times normal after 350 days. Fare and Woodhouse (1963*a*) found that when rats were given maize + 0·5 per cent of cupric oxyacetate alone, the content of copper in the liver increased by 200 times after 380 days, and thus it would seem that giving DMAB in addition results in a lesser storage not apparently affected by the presence in the diet of the other factors which protect against carcinogenesis.

Fig. 4 gives the succinic dehydrogenase activities after 350 days for each dietary group ; the relative values are similar in the case of cytochrome oxidase activity.

### DISCUSSION

In Table II, the results are compared from the various criteria and an overall assessment is made. Small differences have been ignored where they were considered not to be significant taking into account the unavoidable experimental error. The four remaining methods of assessment agree fairly well, and it can be

FIG. 2.

FIG. 2.—Liver nitrogen content, mg. Kjeldahl nitrogen per 100 mg. parenchymatous pulp. Broken line shows lower limit of normal range. Values are shown only where they are below the normal range.

TABLE II.—*Assessment of the Protection Afforded by the Various Additives*

| Diet | Liver. Morphology | Weight ratio | Liver. Nitrogen | Liver. Enzyme activity | Overall |
|---|---|---|---|---|---|
| C . | 2= . | 2= . | 2= . | 3= . | 2= |
| D . | 6= . | 6= . | 6= . | 7 . | 6= |
| E . | 6= . | 6= . | 6= . | 6 . | 6= |
| F . | 2= . | 2= . | 2= . | 3= . | 2= |
| G . | 2= . | 2= . | 2= . | 2 . | 2= |
| H . | 1 . | 1 . | 1 . | 1 . | 1 |
| I . | 5 . | 5 . | 5 . | 3= . | 5 |

In each case, the diet is assigned a number from 1 to 7 (1 = best protection). The " = " signifies that two or more diets cannot be differentiated with regard to a parameter. Diet C contains CuAc, D yeast extract, E beef extract, F CuAc and beef extract, G CuAc and yeast extract, H CuAc and both extracts, and diet I contains both extracts.

stated with confidence that the best protection was afforded by giving the copper salt together with both extracts. Even here, however, the liver showed early signs of carcinogenesis after 300 days, and presumably tumours would develop in a proportion of rats if carcinogen treatment was continued.

FIG. 3. O DIET B ▲DIET D ●DIET E ■DIET I

FIG. 3.—Liver copper content (μg. per g. pulp) of rats receiving diets not supplemented with copper. The interrupted line gives the mean for normal rats and the arrow gives the standard deviation.

The groups D and E gave identical results and showed a significant degree of protection, but rather less than did diet G. It would therefore seem that about 30 mg. of extra copper daily was more effective in this respect than 40 μg. of riboflavin. Other possible prophylactic agents in diets D and E (protein, peptides, amino acids, other vitamins) must either have played little part or else were present in the two extracts in similar amounts, since the same protection was given in each case and both diets contained the same amount of riboflavin.

It is of particular interest that diet I, which contained half quantities of both extracts and which also therefore contained 40 μg. of riboflavin, gave a measurably better degree of protection than did either diet D or diet E. Presumably this must be due to the combined effects of cofactors contained separately in the two extracts.

The protection given by copper acetate was not enhanced by the addition of either extract separately, in contrast with the results when diet H containing additionally both extracts was given. A similar explanation holds presumably in this case also.

Many of these deductions have been based on groups of only 6 rats (groups D–I inclusive). These particular dietary groups are in use at the moment in a further experiment designed to investigate dye binding and riboflavin storage in

FIG. 4.—Liver succinic dehydrogenase activity expressed as cu. mm. $O_2$ at N.T.P. per hour per 100 mg. pulp. After 350 days treatment.

the rat liver. Copper and enzyme assays are not being done, but the ratios of body weight to liver weight and the times of discovery of liver damage and tumours are presented in Table III although the experiments themselves are still in course, having run for only 12 months.

By comparing the results in Tables I and III and Fig. 1, it can be seen that once again diets D and E gave the least protection, diet I somewhat better, the copper-containing diets better again and the diet containing all three the best degree of protection.

Although Maisin and Lambert (1960) were unable to demonstrate protection against DMAB carcinogenesis by " substantial amounts " of copper, they reported that beef liver and certain fractions derived therefrom gave a very good degree of protection. These workers could not account for the potency of their material solely in terms of riboflavin content, and it may be that similar potentiating agents could be responsible for the activities of both beef liver and the combined extracts used here.

TABLE III.—*Times at which Liver Damage and Liver Tumours were First Found and the Ratio of Body Weight to Liver Weight after 350 Days*

| Diet | First detection of liver damage (days) | First detection of liver tumours (days) | Post-mortem ratio of body weight to liver weight |
|---|---|---|---|
| C | 240 | — | 18·0 |
| D | 120 | 276 | 11·6 |
| E | 132 | 290 | 11·9 |
| F | 236 | — | 18·4 |
| G | 222 | — | 17·3 |
| H | 300 | — | 24·7 |
| I | 197 | 338 | 14·0 |

These results are from a separate series of experiments, but are included here to demonstrate the reproducibility of the effects of the diets.

Further experiments are being carried out in these laboratories using cupric oxyacetate as a prophylactic against several liver damaging agents and carcinogens. Protection is given against rat liver lesions induced by the chronic administration of thioacetamide (Fare, unpublished work) and of 3-methoxy-4-dimethylaminoazobenzene and its N-monomethyl derivative, but not against the skin and ear-duct tumours produced by the methoxy dyes (Fare and Howell, 1964). In all these cases, the protective effect of copper acetate may be due to competitive binding with the carcinogen for liver protein sites, and the extracts in the experiments described here may function by increasing the rate of enzymic destruction of the dye, facilitating regenerative protein synthesis to replace material destroyed by DMAB, or, when copper is also fed, by effecting binding of the copper preferentially at the expense of the dye at binding sites in the liver.

### SUMMARY

Albino rats given maize + 0·09 per cent of 4-dimethylaminoazobenzene for 7 months or longer all developed liver tumours.

The addition of 0·5 per cent of cupric oxyacetate gave a better degree of protection than did either a yeast autolysate or a beef extract, and adding either extract to the diet containing the dye and copper did not give enhanced protection.

When the two extracts were given together, their effectiveness was greater than the sum of their separate activities.

When both extracts and copper were given, almost complete protection resulted.

In every case, the degree of protection was measured by the increase in time necessary for chemical and gross morphological changes in the liver to become apparent.

I am indebted to Dr. D. L. Woodhouse for his advice and encouragement throughout this work and to Professor J. W. Orr for helpful suggestions and discussion.

My thanks are due to Marmite Ltd., and Oxo Ltd., not only for generous gifts of materials, but also for help and information so freely given.

A scholarship from the Medical Research Council is gratefully acknowledged. This work was supported by the Birmingham branch of the British Empire Cancer Campaign for Research.

### REFERENCES

FARE, G.—(1964) *Biochem. J.*, **91**, 473.

*Idem* AND HOWELL, J. S.—(1964) *Cancer Res.*, **24**, 1279.

*Idem* AND WOODHOUSE, D. L.—(1963*a*) *Brit. J. Cancer*, **17**, 512.—(1963*b*) *Ibid.*, **17**, 775.

HOWELL, J. S.—(1958) *Ibid.*, **12**, 594.

KENSLER, C. J., SUGIURA, K., YOUNG, N. F., HALTER, C. R. AND RHOADS, C. P.—(1941) *Science*, **93**, 308.

KING, H. J., SPAIN, J. D. AND CLAYTON, C. C.—(1957) *J. Nutrition*, **63**, 301.

MAISIN, J. AND LAMBERT, G.—(1960) 'Biological Approaches to Cancer Chemotherapy'. London (Academic Press Inc.). p. 399.

MILLER, J. A. AND MILLER, E. C.—(1953) *Advanc. Cancer Res.*, **1**, 339.


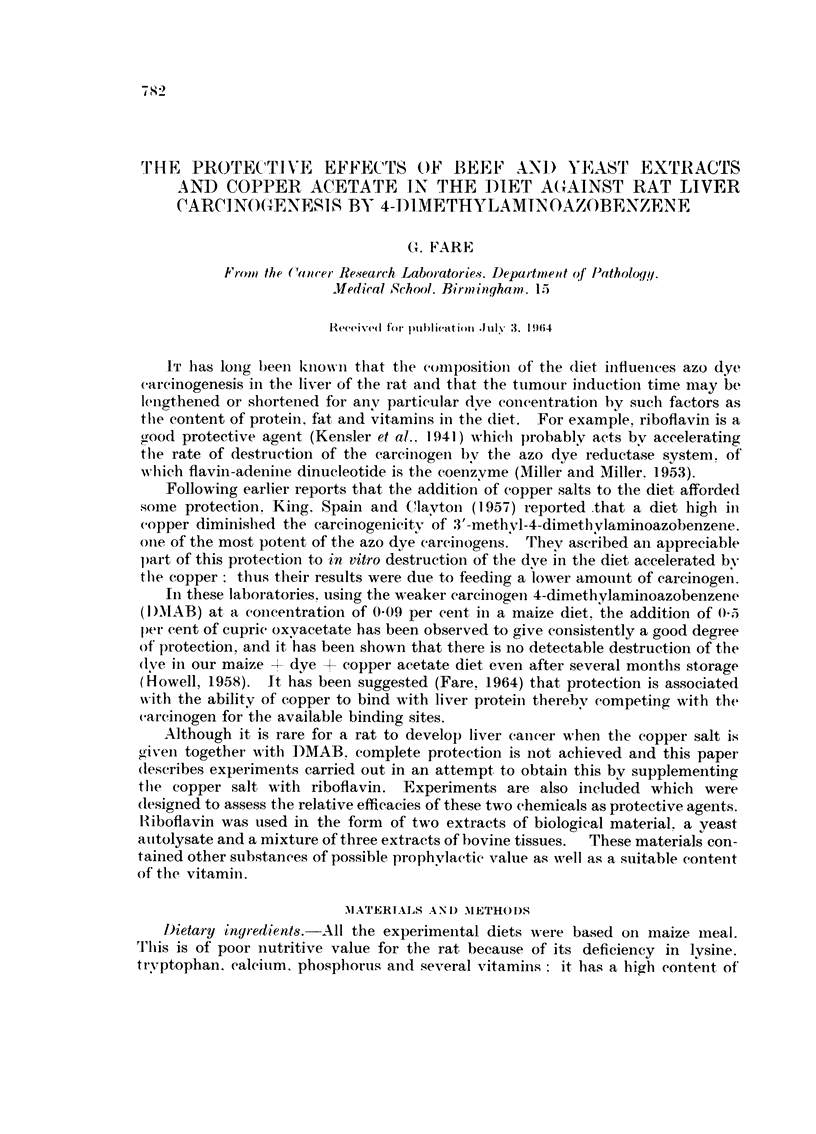

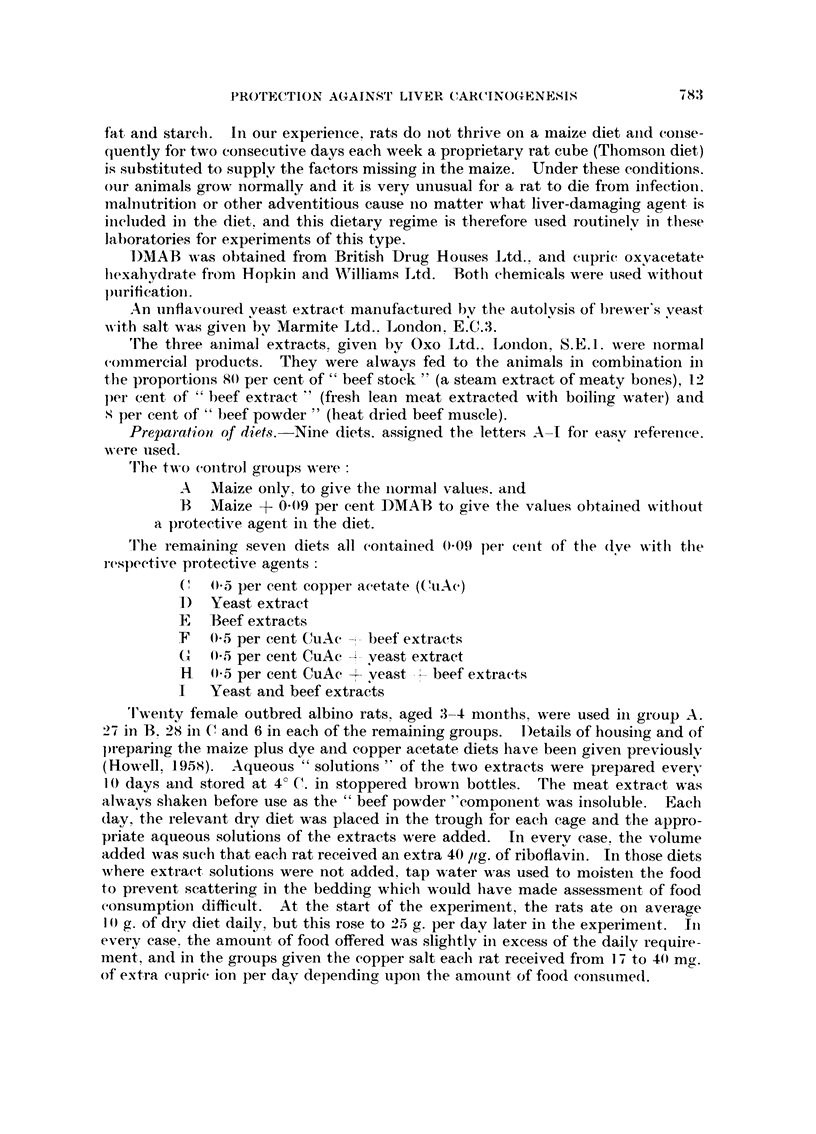

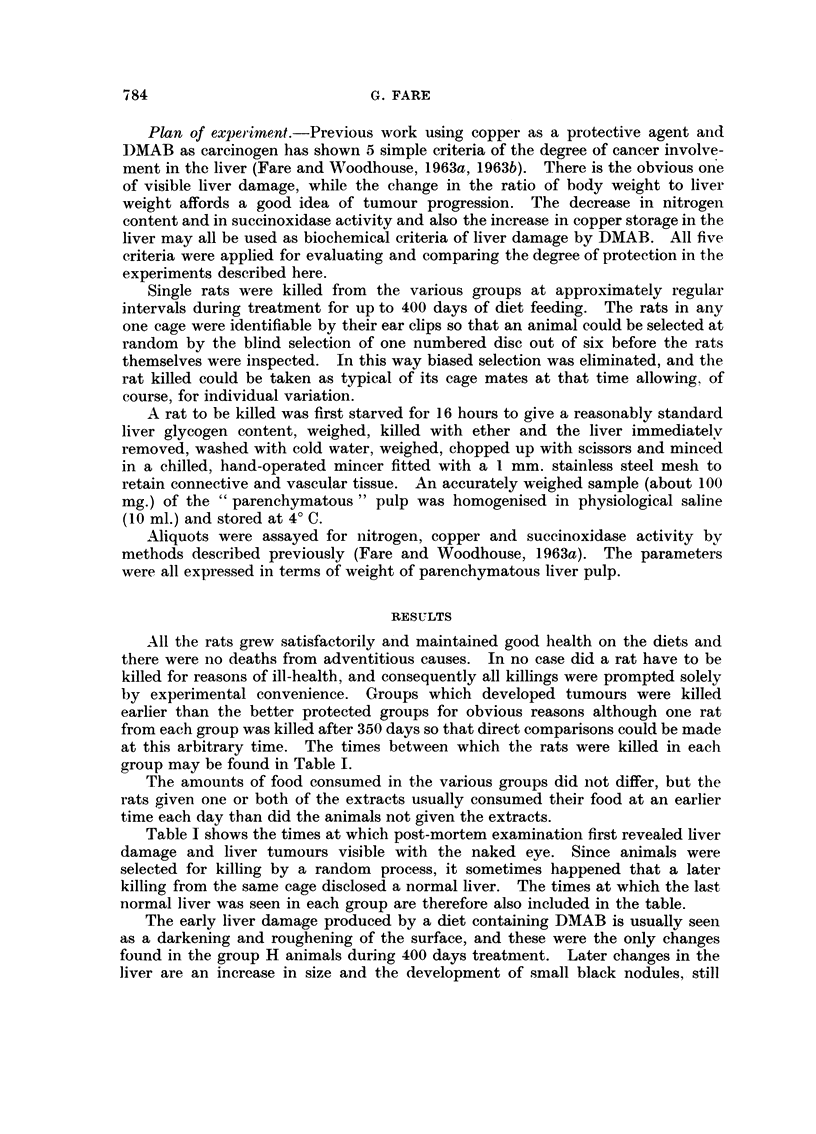

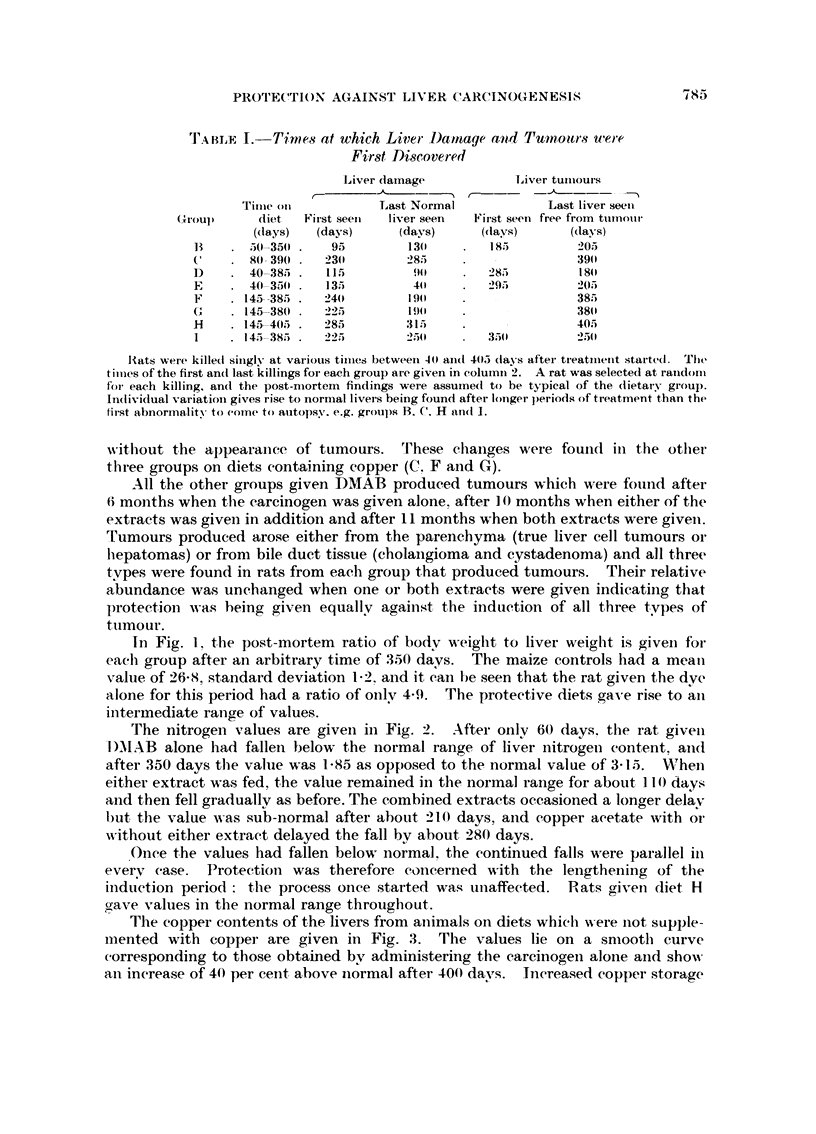

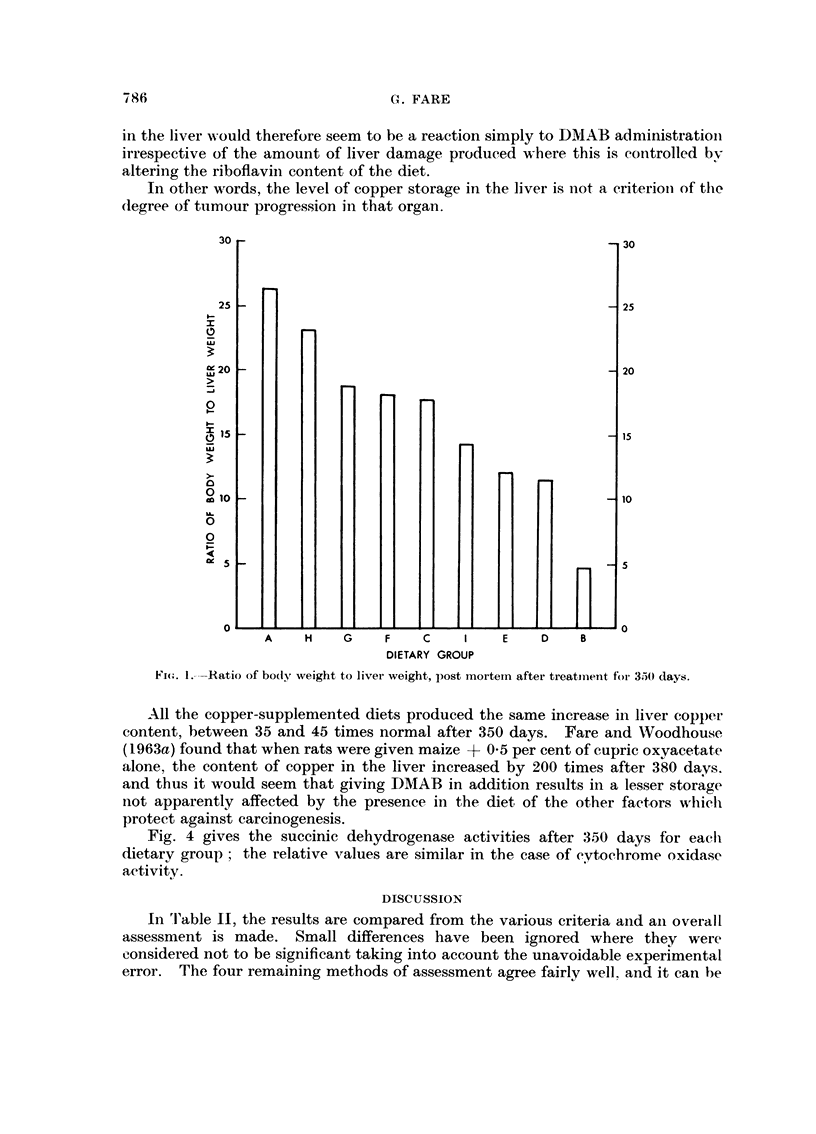

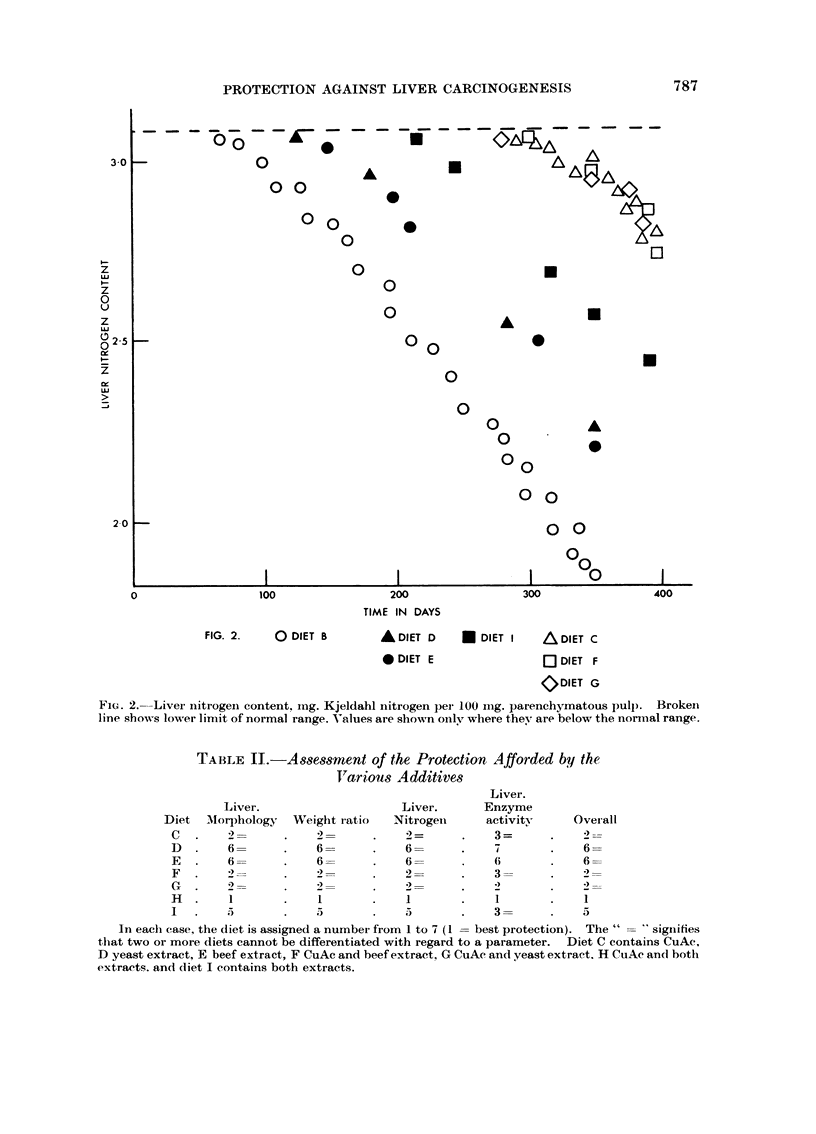

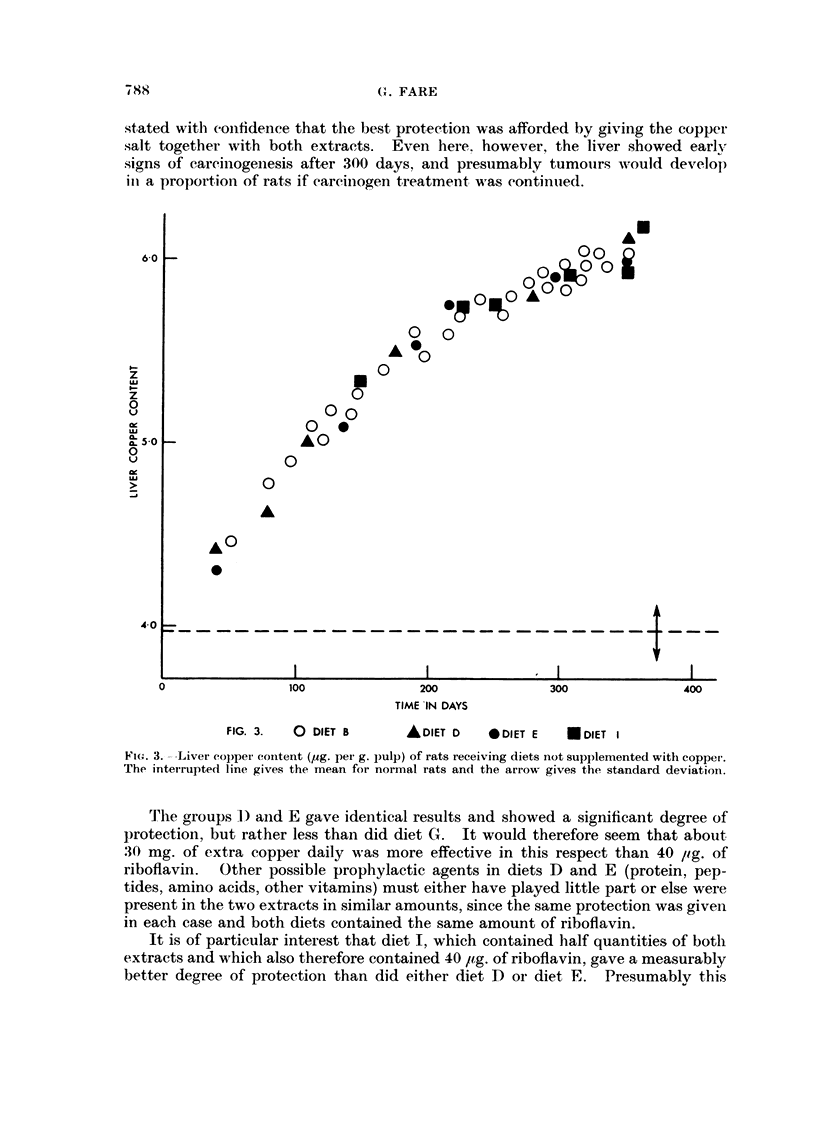

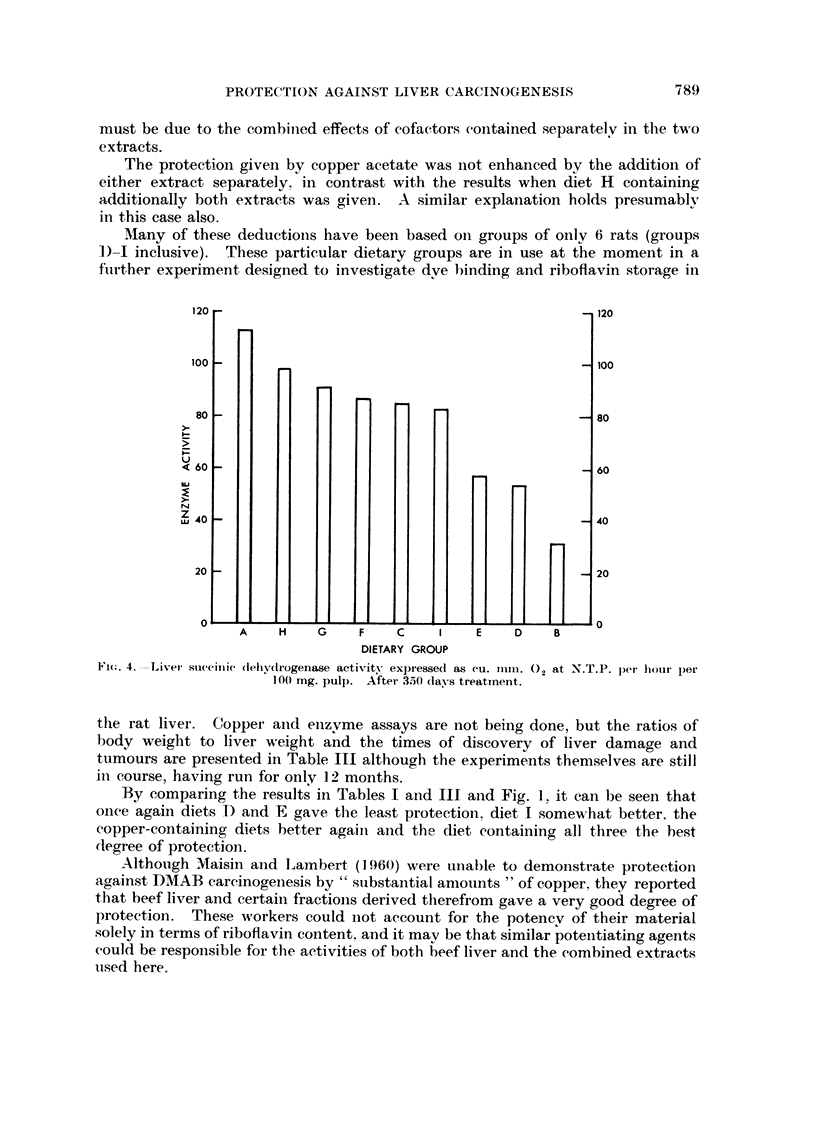

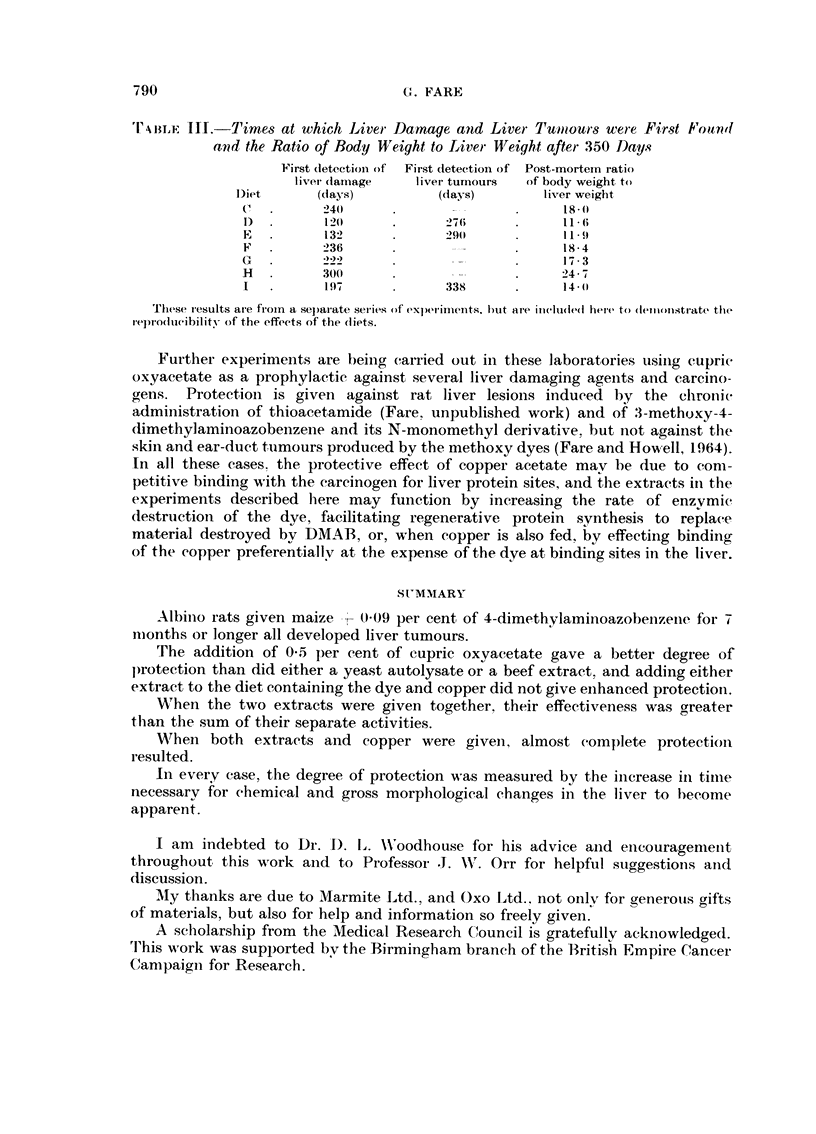

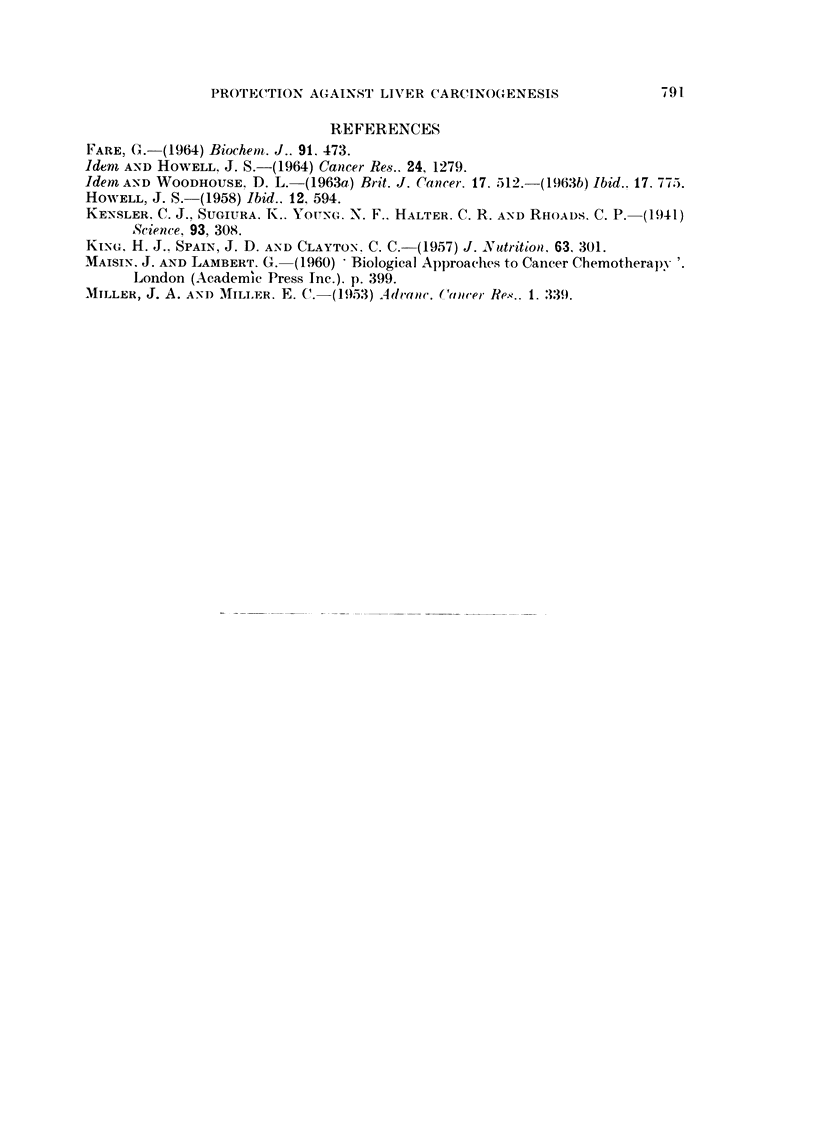


## References

[OCR_00727] Fare G. (1964). The effect of cupric oxyacetate on the binding of azo-dye by protein during the induction of liver tumours in the rat.. Biochem J.

[OCR_00736] KING H. J., SPAIN J. D., CLAYTON C. C. (1957). Dietary copper salts and azo dye carcinogenesis.. J Nutr.

